# Beyond the target area: an integrative view of tDCS-induced motor cortex modulation in patients and athletes

**DOI:** 10.1186/s12984-019-0581-1

**Published:** 2019-11-15

**Authors:** Edgard Morya, Kátia Monte-Silva, Marom Bikson, Zeinab Esmaeilpour, Claudinei Eduardo Biazoli, Andre Fonseca, Tommaso Bocci, Faranak Farzan, Raaj Chatterjee, Jeffrey M. Hausdorff, Daniel Gomes da Silva Machado, André Russowsky Brunoni, Eva Mezger, Luciane Aparecida Moscaleski, Rodrigo Pegado, João Ricardo Sato, Marcelo Salvador Caetano, Kátia Nunes Sá, Clarice Tanaka, Li Min Li, Abrahão Fontes Baptista, Alexandre Hideki Okano

**Affiliations:** 1 Edmond and Lily Safra International Institute of Neuroscience, Santos Dumont Institute, Macaíba, Rio Grande do Norte Brazil; 2 Brazilian Institute of Neuroscience and Neurotechnology (BRAINN/CEPID-FAPESP), University of Campinas, Campinas, São Paulo, Brazil; 3 Universidade Federal de Pernambuco, Recife, Pernambuco Brazil; 4 Núcleo de Assistência e Pesquisa em Neuromodulação (NAPeN), Universidade Federal do ABC (UFABC)/Universidade de São Paulo (USP)/Universidade Cidade de São Paulo (UNICID)/Universidade Federal de Pernambuco (UFPE), Escola Bahiana de Medicina e Saúde Pública (EBMSP), Santo André, Brazil; 5 Department of Biomedical Engineering, The City College of New York of CUNY, New York, NY USA; 6 Center of Mathematics, Computing and Cognition (CMCC), Universidade Federal do ABC (UFABC), Alameda da Universidade, 3 - Anchieta, Bloco Delta – Sala 257, São Bernardo do Campo, SP CEP 09606-070 Brazil; 7 Aldo Ravelli Center for Neurotechnology and Experimental Brain Therapeutics, Department of Health Sciences, International Medical School, University of Milan, Milan, Italy; 8 School of Mechatronic Systems Engineering, Simon Fraser University, Surrey, British Columbia Canada; 9 Department of Physical Therapy, Sackler Faculty of Medicine and Sagol School of Neuroscience, Tel Aviv University, Tel Aviv, Israel; 10 Graduate Program in Physical Education. State University of Londrina, Londrina, Paraná, Brazil; 11 Laboratory of Neuroscience (LIM-27), Universidade de São Paulo, São Paulo, São Paulo Brazil; 12 Department of Psychiatry and Psychotherapy, University Hospital, LMU Munich, Munich, Germany; 13 Graduate Program in Rehabilitation Science, Universidade Federal do Rio Grande do Norte, Santa Cruz, Rio Grande do Norte Brazil; 14 Escola Bahiana de Medicina e Saúde Pública, Salvador, Bahia Brazil; 15 Laboratório de Investigações Médicas-54, Universidade de São Paulo, São Paulo, São Paulo Brazil

**Keywords:** Neuromodulation, Non-invasive brain stimulation, Motor rehabilitation, Motor learning, Motor performance, Sport, HD-tDCS, tsDCS, ctDCS, TMS-evoked potential, Connectivity

## Abstract

Transcranial Direct Current Stimulation (tDCS) is a non-invasive technique used to modulate neural tissue. Neuromodulation apparently improves cognitive functions in several neurologic diseases treatment and sports performance. In this study, we present a comprehensive, integrative review of tDCS for motor rehabilitation and motor learning in healthy individuals, athletes and multiple neurologic and neuropsychiatric conditions. We also report on neuromodulation mechanisms, main applications, current knowledge including areas such as language, embodied cognition, functional and social aspects, and future directions. We present the use and perspectives of new developments in tDCS technology, namely high-definition tDCS (HD-tDCS) which promises to overcome one of the main tDCS limitation (i.e., low focality) and its application for neurological disease, pain relief, and motor learning/rehabilitation. Finally, we provided information regarding the Transcutaneous Spinal Direct Current Stimulation (tsDCS) in clinical applications, Cerebellar tDCS (ctDCS) and its influence on motor learning, and TMS combined with electroencephalography (EEG) as a tool to evaluate tDCS effects on brain function.

## Introduction

Transcranial electrical stimulation has recently attracted considerable scientific interest due to its ability to modulate brain functioning. From a historical perspective, ancient Greek philosophers Plato and Aristotle were both aware of the torpedo fish electrical discharges capacity to elicit therapeutic effects [1, 2]. The use of a live torpedo fish on the scalp to cure headaches might indeed be classified as an early form of transcranial direct current stimulation (tDCS). This practice consists in applying a direct current in a transcranial way, as contrasted with intracranial way, and with a putative brain activity modulation effect. The fish electrical stimulation was used for the treatment of epilepsy, demonic possessions, headaches, and even gout for over 10 centuries [2, 3].

Currently, tDCS devices apply a weak direct electrical current (0.5–2 mA, typically power by a 9 V battery) through two or more electrodes placed on the scalp, typically for a relatively long period of time (e.g., 20 min) to facilitate or inhibit spontaneous neuronal activity. The stimulation facilitates or inhibits spontaneous neuronal activity putatively resulting in cortical excitability modulation [4–7] and neuroplastic reorganization [8–11]. tDCS has been used in neuropsychiatric [12–14] and neurological disorders [15–19], modulation of autonomic nervous system [20–23], appetite [24–26], energy expenditure [27], motor performance [21, 28, 29] and motor learning [8, 30–33]. More recently, a high-definition-tDCS (HD-tDCS) was developed with arrays of smaller “high-definition” electrodes to increase brain modulation accuracy [34, 35]. Delivery of HD-tDCS is capable of inducing significant neurophysiological and clinical effects in both healthy subjects and patients [36].

**Fig. 1:**
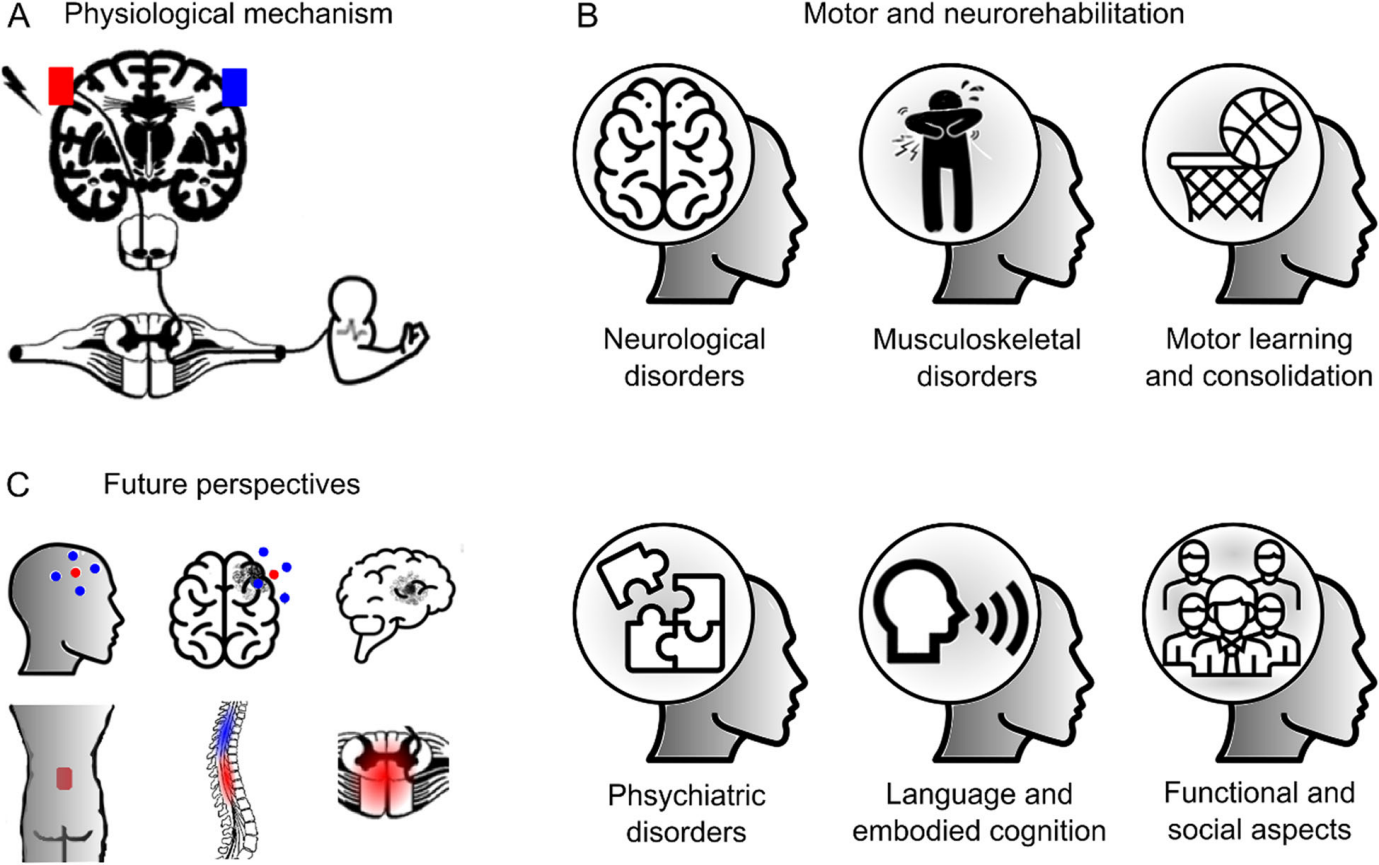
Many different studies have shown tDCS beneficial results on motor rehabilitation, but very few have discussed the potential integrative effect of tDCS beyond the target area. This figure depicts an overview from: **a**physiological mechanisms, **b**motor and neurological rehabilitation to **c** futures perspectives with high definition tDCS. The growing scientific literature results in many different disorders supports the integrative involvement of researchers to ultimately improve the quality of life of thousands of patients around the world

Therefore, the current paper aims to review, based on an integrative approach, the current state of knowledge focused on the following research topics: (1) Physiological basis and mechanisms of tDCS in motor rehabilitation and motor learning; (2) tDCS as a motor rehabilitation in neurological disorders; (3) tDCS as a form of motor rehabilitation in musculoskeletal disorders; (4) tDCS as a tool to counteract maladaptive plasticity in chronic musculoskeletal pain; (5) facilitation of motor learning and consolidation by tDCS in patients and athletes; (6) underappreciated motor cortex stimulation for psychiatric disorders; (7) language and embodied cognition; (8) functional and social aspects; (9) High-definition tDCS (HD-tDCS) on neurologic disease, pain relief and motor learning/rehabilitation. (10) Transcutaneous Spinal Direct Current Stimulation (tsDCS) on clinical applications; (11) Cerebellar tDCS (ctDCS) and its influence on motor learning; and (12) TMS combined with electroencephalography (EEG) as a tool to evaluate tDCS effects on brain function. These topics are summarized in the Fig. 1.

## Physiological basis and functional connectivity of tDCS in motor rehabilitation and motor learning

### Mechanisms of tDCS in motor rehabilitation and motor learning

**Fig. 2:**
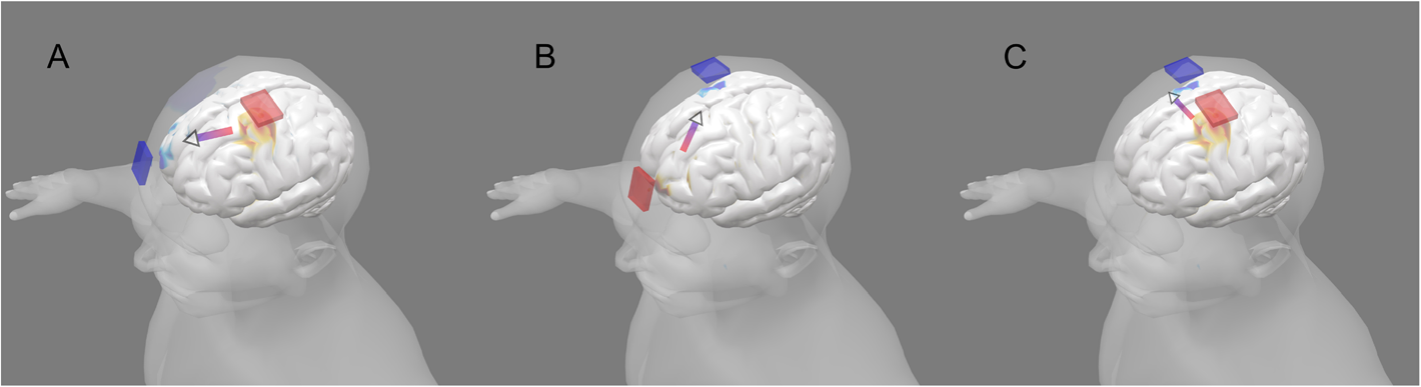
Examples of tDCS montage and the current flow to stimulate left primary motor cortex (M1). **a** Anodal stimulation delivered on left M1 depolarizes the resting membrane potential and increases neuronal excitability. **b** Cathodal stimulation on right M1 hyperpolarizes the resting membrane potential and decreases neuronal excitability. **c** Simultaneous stimulation of left M1 (anode - increasing excitability) and right M1 (cathode - decreasing excitability)

tDCS generates low intensity-sustained current (electric field) in the brain [35, 37, 38]. There are two related mechanisms of tDCS that support its use in motor rehabilitation: modulation of neuronal excitability and plasticity (for a general review of tDCS mechanisms see [39]. For decades, it has been established in animal models that direct current stimulation (DCS) can produce polarity-specific changes in neuronal excitability; “anodal” and “cathodal” polarities provide increasing and decreasing excitability, respectively [40] (Fig. 2). When DCS is sustained for several minutes, animal [41, 42] and canonical human neurophysiology studies using TMS [43] have demonstrated changes in neuronal excitability that are persistent for minutes after termination of stimulation. Animal models have further linked long-term changes in excitability with synaptic plasticity (e.g., long-term potentiation; LTP [8, 44–46] while clinical trials of tDCS have investigated lasting changes following repeated sessions. The modulation of excitability, measured during or acutely after stimulation, and plasticity based on markers of LTP or long-term monitoring, are related. The application of tDCS in neurorehabilitation is not surprising, since it can be used to increase or decrease brain function and learning [47–50], and it is considered safe and well-tolerated [51, 52]. Evidence from DCS clinical trials is further supported by animal models of injury recovery [39, 53–57].

The biophysics and nuance of using DCS to produce lasting changes in brain function have been extensively studied. The cellular targets of DCS include the soma of pyramidal neurons [58, 59], axon terminals/synapses [60–62] and dendrites [45]. In each of these cases, membrane polarization of the cellular targets by current flow is the initial cellular mechanism of action. One key nuance is that there is no such thing as an “only depolarizing” or “only hyper-polarizing” mode of DCS; rather, every neuron has compartments that are depolarizing and compartments that are simultaneously hyperpolarized during DCS [60, 63]. Changing the polarity of stimulation reverses the polarization in each given compartment. For example, it is correct to say that “anodal” DCS will depolarize the somas of most cortical pyramidal neurons while recognizing that other compartments of those neurons and of neighboring cells will be simultaneously hyperpolarized [59]. Despite the complex polarization pattern, there can be significant directed changes in function (as noted above), but the role of polarity may vary with nuance in underlying brain activity [45].

A further key nuance of DCS is “functional targeting” [64]. Because tDCS may be too low intensity to generate activity de novo, the idea is that specific brain networks become activated by a task (e.g. rehabilitation training) and, because they are already active, these networks (and not others) become more sensitive to tDCS [39]. For example, only synapses already undergoing plasticity would be modulated by DCS, while inert synapses would not be activated or modulated [45]. This feature can be a virtue since it supports exquisite selectivity: only those brain regions activated by a task would be susceptible to be modulated by tDCS. These results also explain the dependence of tDCS on brain state [64–68], which can be understood not as a limitation but rather a factor to control and leverage [69].

The flow of electrical current through the brain changes by the presence of a lesion [70–73] or injury [74]. Computational models of current flow can be used to account for and optimize current delivery in such cases [75]. While which current flow pattern is best suited for a given clinical or rehabilitation indication is still an open question (relating to the mechanisms of DCS), the current flow models are already validated [76].

Alternative or complementary mechanisms of DCS include modulation of oscillations [67, 77], glial function [78, 79], vascular function [80, 81], growth and mobility [82, 83] or neurogenesis [84, 85]. In addition, over a decade of systematic research in animals and human trials have demonstrated differences in the dose and brain-state dependent aspects of tDCS modulation, particularly in the motor system. For example, changing the montage [6, 34, 86], polarity [66], intensity [87, 88], duration, concomitant medication [89], or task may qualitatively change outcomes [9]. It is important to recognize that the decades of work on DCS and ongoing emerging insights into the nuances of stimulation not necessarily a deficiency of understanding tDCS. Conversely, it reflects that tDCS is a technique far better characterized than most interventions [90–92] and the inherent complexity of brain function. In the context of neurorehabilitation, ongoing research is thus not directed to the general plausibility of enhancement by tDCS (as a tool to modulate excitability and plasticity) but rather specifically how to account for these nuances in order to optimize rehabilitation outcomes [93–95] including reducing variability in responsiveness [96–99].

## tDCS and brain connectivity on the motor cortex

**Fig. 3:**
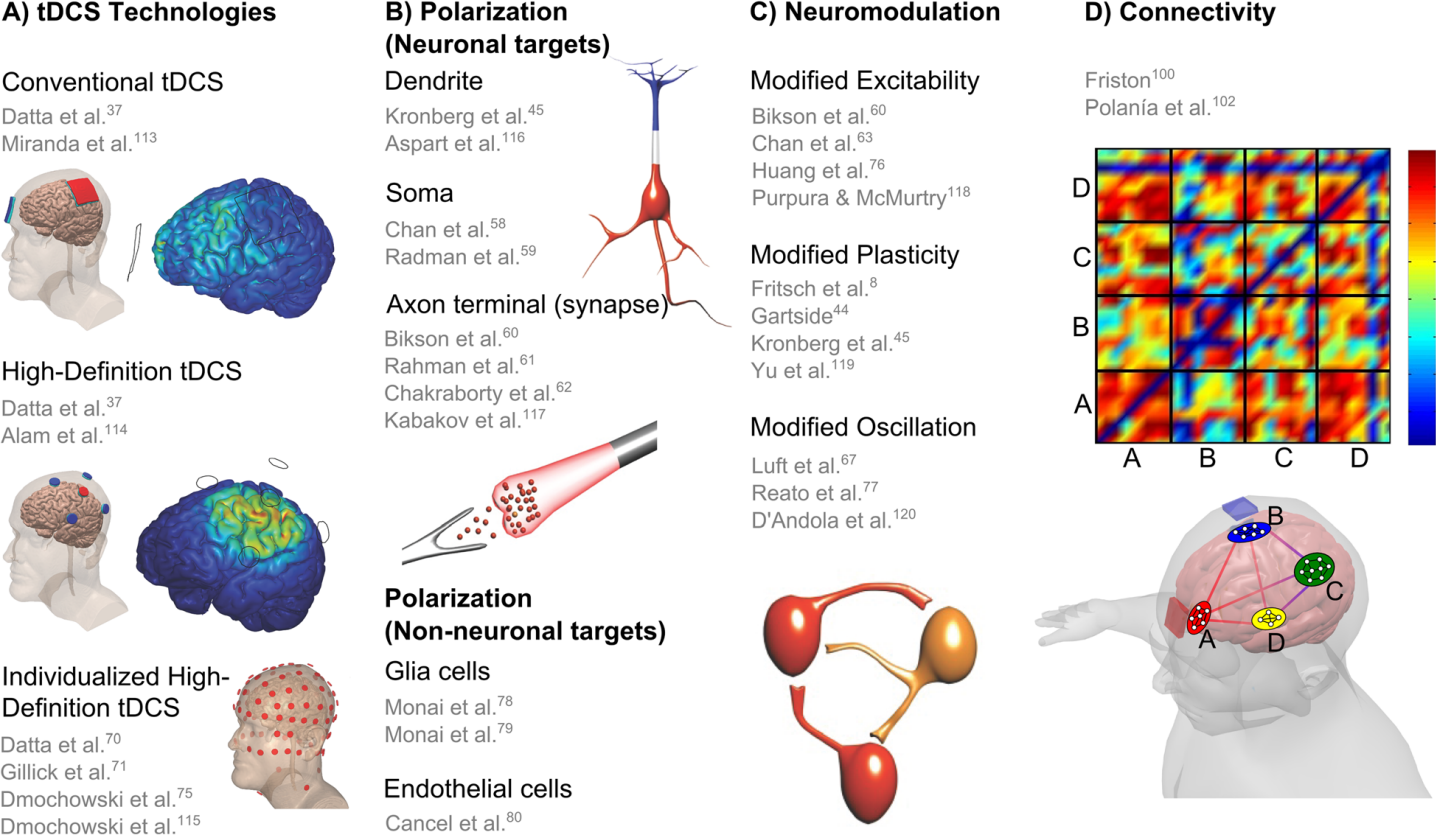
Physiological basis and mechanisms of tDCS. **a** Several studies in the last ten years support tDCS technologies with beneficial results using conventional tDCS [37, 113], High-Definition tDCS [37, 114] and individualized High-Definition tDCS [70, 71, 75, 115]. **b** The current flow direction affects differently dendrite [45, 116], soma [58, 59], axon terminal [60–62, 117], glia [78, 79] and endothelial cells [80]. Anodal stimulation hyperpolarizes apical dendritic layer (blue) and depolarize soma (red) of pyramidal cortical neurons. **c** The resultant tDCS effects reported are related to modified excitability [60, 63, 76, 118], neuroplasticity [8, 44, 45, 119] and neural network oscillation [67, 77, 120]. **d** Simulation of four brain networks during tDCS with a connectivity (or adjacency) matrix between a given pair of regions by connectivity strength [100, 102]

Brain connectivity research focuses on anatomical pathways, interactions and communications between different regions of the central nervous system. The connectivity analysis based on brain activity can be undirected or directed and classified as functional, if it measures the statistical dependence of signals, or effective if it takes into consideration the causal relationship between signals. The regions of interest can be defined in micro- or macro-scale levels and their interaction can be considered as static or dynamic. Brain connectivity methods have been substantially applied to the study of the motor cortex, extracting new features from resting state, motor and imagery tasks. The underlying networks are built using EEG, functional magnetic resonance imaging (fMRI) and functional near-infrared spectroscopy (fNIRS) data and then assessed through functional connectivity (FC) or effective connectivity (EC) measures, for healthy and pathological subjects [100, 101]. See Fig. 3d for an example of brain networks activation during tDCS and the respective connectivity matrix visualization.

The first report of tDCS effects on motor cortical connectivity is the work of Polaina and collaborators [102]. They applied anodal tDCS over M1 in resting state and during motor tasks performed by healthy subjects. The FC from the EEG signals in different frequency bands were calculated and compared before and after the stimulation. They observed significant intrahemispheric and interhemispheric connectivity changes in all bands and conditions. Specifically, in theta and alpha bands, FC increased between frontal and parietal-occipital areas after the stimulation, during hand movements, evidencing robust tDCS-induced alterations in the sensory-motor brain network. Further studies analyzed the brain connectivity from EEG recordings after the stimulation of the motor cortex. Hordacre and colleagues [103] investigated the anodal tDCS in chronic stroke patients on the lesioned M1. The FC analysis showed stronger connectivity between ipsilesional parietal cortex and contralesional frontotemporal cortex, in the alpha band, associated with the increase of corticospinal excitability following the stimulation. This association was not observed in sham stimulations and suggests FC as a biomarker of therapy response. Baxter and coauthors [104] studied the effects of anodal tDCS on the motor cortex connectivity during motor imagery tasks. The target was the left sensorimotor cortex and they calculated the EC between EEG channels related to the frontal and parietal regions. Comparing pre- and post-stimulation conditions, the findings in the alpha band reveal different correlates in a task-specific manner. During right-hand imagination, EC increased from the ipsilateral PMC and contralateral sensorimotor cortex to the target area. In addition, during left-hand imagination, EC increased from the target area to multiple regions across the motor cortex. The results showed a task-specific modulation between tDCS and brain network organization. Gaxiola-Tirado and collaborators [105] examined the stimulation effects during motor imagery tasks. They found strong FC in alpha and beta bands between central channels, following tDCS on the lower limbs. In the sham group, they noticed more random connections in these regions.

An increasing number of studies have considered resting-state functional magnetic resonance imaging to understand the connectivity pattern shifts in the default mode network observed after tDCS. Sankarasubramanian and colleagues [106] reported a Thalamocortical networks study focused on the pain matrix. They demonstrated that anodal M1 tDCS increased FC between ventroposterolateral area and sensorimotor cortices and also between motor dorsal and motor cortices. The findings suggest that M1 stimulation modulates FC of sensory networks. Lefebvre et al. [107] showed that a single session of dual-tDCS combined with motor skill learning increases FC between M1 and PMd of the damaged hemisphere in chronic stroke patients, supporting the hypothesis that changes in FC correlate with recovery. Chen and coauthors [108] analyzed FC in individuals with stroke. The connectivity increased between ipsilesional motor cortex and contralesional premotor cortex after tDCS in motor rehabilitation, suggesting that the activation of interactions between motor and premotor cortex might be beneficial for stroke motor recovery. Sehm and colleagues [109] studied different setups of tDCS over the M1. The bilateral and unilateral M1 tDCS induced a decrease in interhemispheric FC during stimulation and the bilateral M1 tDCS induced an increase in intracortical FC within right M1 after the intervention. Depending on the tDCS montage, the connectivity analysis revealed different effects in M1 processing and can explain the induced changes in motor performance and learning from the perspective of the neural networks modulation. Rosso et al. [110] examined brain connectivity after cathodal tDCS applied to the right inferior frontal gyrus, before a picture-naming task performed in healthy individuals. They found greater FC between the right Broca’s area and the supplementary motor area (SMA) and these findings were correlated to the improvement of learning abilities, in the sense that subjects named pictures faster after cathodal relative to sham tDCS.

Besides EEG and fMRI data, tDCS effects on brain connectivity can also be examined based on hemodynamic changes. For instance, Yan et al. [111] observed the resting state fNIRS and showed that the FC between intracortical regions decreased during anodal tDCS in the motor cortex indicating a relationship between brain network changes due to the stimulation and the hemodynamic responses.

There is extensive literature investigating electrical brain stimulation and FC. Therefore, future work should investigate more correlates between tDCS and directed brain interactions through EC measures, in different frequency bands, including cross-frequency causality. These time-varying causal brain networks captured by EC can modulate power spectra and behavioral responses [112], opening new possibilities, advancing the state of art of the tDCS therapy on the motor cortex and extending the knowledge on the effects beyond the target area. Figure 3 summarizes the physiological basis and mechanisms of tDCS.

## tDCS as a motor neurorehabilitation tool in neurological disorders

Neurological disorders resulting from injury or disease of the nervous system are a significant cause of disability and death worldwide [121]. Patients with disability due to neurological conditions have significant socioeconomic implications due to long-term functional and psychosocial issues, and requirement for specialized rehabilitation services [122–124]. Advances in the understanding of brain function, recovery from injury and neuroplasticity have provided a basis for developing new technologies that are slowly becoming part of neurorehabilitation approaches, especially the increasing application of tDCS [125–127]. This review summarizes the applications of DCS in the most common neurological disorders investigated in tDCS trials.

### Stroke

Rehabilitation of motor function after stroke is the most thoroughly studied clinical application of tDCS in neurorehabilitation. Beneficial effects of tDCS on post-stroke rehabilitation have been reported in meta-analyses concerning the upper [128–131] and lower-limb functions [132] and mobility [49, 132, 133].

**Fig. 4:**
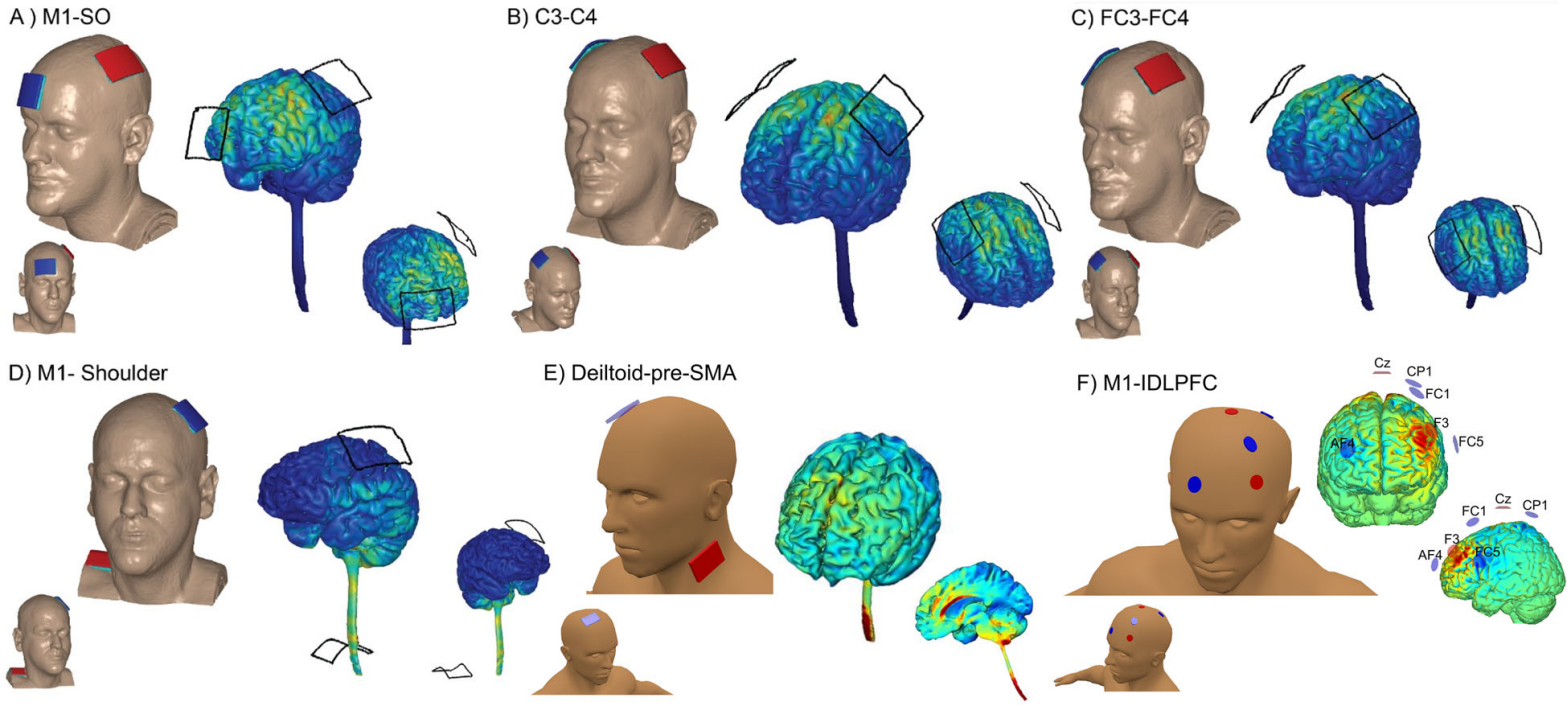
Examples of electrode montage. **a** Spinal Cord Injury [137]: 5x7 cm; 2 mA; 20 min; 10 sessions; the anodal electrode placed over C3/C4 contralateral to the targeted arm and the cathodal electrode located over contralateral supraorbital area. Musculoskeletal disorders/Pain [18, 138]: 5x7 cm; 2 mA; 20 min; anodal C3/cathodal Fp2; 5 sessions. Motor learning [139]: 5x5 cm; 1 mA; 20 min; 5 sessions; the anodal electrode placed over a presumed “target” (eg.: left M1 to target right upper limb, C3), with the cathodal electrode located over the contralateral supraorbital region (eg.: right supraorbital area, Fp2). **b** Stroke [140, 141]: 4x4 cm or 5x7 cm; 1.5 or 2 mA; 30-40 min; 5-10 sessions; dual tDCS where the anodal is placed over ipsilesional (eg.: left M1) and cathodal over contralesional hemisphere (eg.: right M1); Dystonia [142]: 5x7 cm; 2 mA; 20 min; 1 session; simultaneous inhibitory and excitatory stimulation on M1 (the cathodal electrode on the affected M1 and the anodal electrode on the unaffected M1); Traumatic Brain Injury [143]: 2x2 cm; 1.5 mA, 15 min; 24 sessions (3 days/week); the anodal electrode placed over the ipsilesional M1 and the cathodal electrode over the contralesional M1. **c** Language [144]: 5x7 cm; 2 mA; 20 min; the cathodal placed at FC3 and the anodal at FC4. **d** Language [145]: 5x7 cm; 2 mA; tDCS started 4 min before the beginning of the task and was delivered for the whole course of the task execution (about 2 min); the cathodal electrode positioned over the left M1 and the anodal electrode placed on the skin overlying the left shoulder region. **e** Psychiatric disorders (Obsessive-compulsive disorder) [146, 147]: 5x5 cm; 2 mA; 20 min; 10 sessions [148]; or 5x5 cm; 2 mA; 30 min; 20 sessions [149]; cathodal placed bilaterally over the SMA and the anodal positioned in the deltoid. **f** Parkinson disease [150]: array of 6 Ag/AgCl electrodes/“Pi-electrodes” of 3 cm^2^ contact area; 20 min; left DLPFC and M1 (multi-target) determined according to the 10–20 EEG system

Based on the model of post-stroke abnormal interhemispheric inhibition [134, 135], three different montages of stimulation to improve motor recovery are commonly used: anodal tDCS (a-tDCS) over the ipsilesional hemisphere, cathodal tDCS (c-tDCS) over the contralesional hemisphere, and dual tDCS where the anode is placed over ipsilesional and cathode over contralesional hemisphere simultaneously [17, 47, 52]. These three montages are supposed to help to normalize the balance of transcallosal inhibition between both hemispheres resulting in improved motor function [136]. However, dual montage (electrode size: 4 × 4 cm or 5 × 7 cm; 1.5 or 2 mA; 30–40 min; 5–10 sessions; Fig. 4b) [140, 141] seems to be superior in reducing motor impairment when compared with anodal tDCS (a-tDCS) or c-tDCS polarity [129]. Nevertheless, tDCS application guided by imbalanced interhemispheric inhibition may be inappropriate in patients with greater cortical damage and more severe motor impairment [125]. fMRI studies demonstrated that an increased contralesional cortical activation may be an adaptive reorganization in severely affected patients [151, 152]. Therefore, the choice of tDCS montage should take each individual patient’s motor functional network into consideration.

Apart from M1 [153–157], other areas such as the SMA [158], primary somatosensory cortex (S1) [159] and premotor cortex (PMC) [160] and cerebellum [50, 161, 162] have been targeted in tDCS studies for stroke motor rehabilitation. Overall, patients in acute [155, 163], subacute [164] and chronic phase [156, 157, 161] have shown improvement in motor impairment after tDCS. A previous meta-analysis reported that tDCS showed a more significant effect size on motor recovery in chronic stroke when compared to acute stroke [129]. When combined with conventional treatment, tDCS can reduce motor impairment in patients with stroke more than motor training isolated [141]. Stimulation has been applied before [153, 154, 157], during [155, 156, 164] and after motor training [165, 166]. Currently, there is insufficient evidence for recommending specific targeted cerebral areas, stroke phase, type of combined therapy and order of stimulation/therapy application for all patients. The magnitude of tDCS effect on stroke motor recovery appears to be influenced by multiple factors such as stroke severity and chronicity, lesion size and location, and cortical tract integrity [52, 166]. Future research should focus on developing the personalized tDCS protocol based on individual patient factors to lead to better motor recovery.

### Parkinson disease (PD)

Advances in the potential therapeutic effects of repetitive transcranial magnetic stimulation (rTMS) [167, 168] have encouraged the use of tDCS as an alternative therapy in PD. Although systematic reviews have not reported the benefit of tDCS for PD motor rehabilitation [92, 169, 170], nevertheless preliminary studies have suggested that tDCS could ameliorate bradykinesia [171], freezing of gait [150, 172], balance and functional mobility [173–177]. However a decrease in PD motor performance was reported [178] and pointed out essential aspects, such as methodological variability among studies, participant characteristics, tDCS protocols, stimulation target, outcome measures, and study design to support congruent findings and conclusive evidence in future reviews.

tDCS studies in PD motor function used distinct stimulation targets, such as the M1 [172], SMA [174, 175], cerebellum [179] and dorsolateral prefrontal cortex (DLPFC) [180, 181]. Other studies used simultaneous stimulation target for multiple cerebral areas [150, 171, 177]. A multitarget stimulation (Fig. 4f) provided a more significant benefit when compared to a single target [150]. Most of these PD therapeutic studies used a-tDCS montage [92], and only few studies investigated tDCS effects combined with conventional therapy in PD [173–175, 182, 183]. Kaski et al. [173] and Costa-Ribeiro [174] demonstrated that the combination of tDCS and motor training improves gait performance more than the training itself. In contrast, Manenti et al. [182] and Schabrun et al. [183] found a non-significant benefit of tDCS combined with motor training. It should be emphasized that tDCS does not replace antiparkinsonian drug, but complements the therapy. As tDCS-induced plasticity is dependent on the dopamine concentration [184], a low dopamine level can impair the tDCS effect [185]. Therefore, future innovative studies should consider the optimal dopamine concentration during tDCS therapy.

### Dystonia

Currently, the beneficial effects of tDCS on motor rehabilitation in dystonia are modest and highly speculative since few studies, is most case reports or small case series, have suggested a potential therapeutic role of the technique [186–190]. Considering that increased excitability or loss of inhibition at multiple levels within and among cortical motor areas was reported in dystonia [191, 192], a possible therapeutic strategy would be to increase the inhibitory cortical drive. Indeed, inhibitory low-frequency rTMS over M1 decreased writing pressure in patients with focal hand dystonia [193]. A similar beneficial effect was obtained when c-tDCS was applied for 5 days over bilateral motor/premotor areas in two musicians with focal hand dystonia [189]. However, failures of c-tDCS to improve fine motor control in writer’s cramp [194] and musicians cramp patients [195, 196] were reported after short intervention period (1–3 sessions). Simultaneous inhibitory and excitatory stimulation on M1 (electrode size: 5 × 7 cm; 2 mA; 20 min; 1 session; c-tDCS on the affected M1 and a-tDCS on the unaffected M1; Fig. 4b) combined with sensorimotor training for 5–10 sessions seems also promising for therapeutic purposes in dystonia [142, 188]. Furuya et al. [142] reported that tDCS fails to improve fine motor control when stimulation is applied without motor training (during rest). Cerebellum has also been a target of tDCS studies in dystonia; however, the results are still contradictory findings [187, 197]. Large clinical trials with multiple-sessions are still required to elucidate the therapeutic role of tDCS on neurorehabilitation of dystonia and to implement it in clinical practice.

### Spinal cord injury (SCI)

Very few studies have examined the effects of DCS in improving motor functions after SCI [198]. Evaluations through multiple-sessions have shown improvement in hand [137] and gait function [199] when stimulating M1 with a-tDCS (electrode size: 5 × 7 cm; 2 mA; 20 min; 10 sessions; the anodal electrode placed over C3/C4 contralateral to the targeted arm and the cathodal electrode located over contralateral supraorbital area; Fig. 4a). Although Kumru et al. [200] found no benefit of combining tDCS with motor training, others studies suggest that pairing tDCS with motor training provides an advantage in improving motor function in individuals with SCI [137, 199, 201]. tsDCS, a promising noninvasive stimulation of central nervous system through a direct current over the spinal cord, emerged as an innovative tool [202]. In healthy individuals, although still debatable [203], tsDCS have been suggested to modulate spinal networks [204, 205]. Therefore, it is expected that tsDCS, modulates spinal function, and motor outcomes in subjects with SCI. Indeed, the findings of Hubli et al. [206] have shown that anodal tsDCS can modulate spinal neuronal circuitries after SCI. Powell et al. [207] have shown that cathodal tsDCS can increase corticospinal excitability contralateral to the reference electrode and decrease corticospinal excitability ipsilateral to the reference electrode. Further studies are needed to understand the extent to which tsDCS can be a complementary treatment to improve motor function in SCI patients.

### Multiple sclerosis (MS)

Over the recent years, the effects of tDCS have been assessed on various MS-related complications including sensory and motor deficit [208–210], spasticity [211], pain [212, 213], fatigue [214–216] and cognitive disorders [217]. Among these, fatigue is the symptom more frequently addressed for tDCS therapeutic studies [218]. Overall, these studies suggest that the application of a-tDCS for 5 consecutive days could decrease fatigue symptoms, but the stimulation site differs among studies, and positive effects were found when tDCS was applied over bilateral S1 [216, 219] or M1 [214]. Over left DLPFC, beneficial [220, 221] or no effect [215] of tDCS was reported. Regarding motor performance, there is speculation about the possibility of tDCS having therapeutic potential but based only on a few single-session studies [208, 210]. More significant therapeutic effects are expected from the application of multiple tDCS sessions in coming studies.

In summary, tDCS probably helps the brain to establish new patterns of activity that support functional recovery. Despite the challenge in drawing a definitive conclusion for all neurological disorders, tDCS has emerged as a promising therapeutic tool for motor neurorehabilitation. However, the successful implementation of tDCS in clinical practice will rely on identifying biological markers which can predict responders and on determining optimal stimulation protocols that take individual patient factors into account. In addition, the rationale for the use of tDCS in neurorehabilitation settings is to provide additional benefit beyond conventional therapy (i.e., to offer an adjunctive approach for patients with neurological disorders).

## Traumatic brain injury (TBI)

TBI can cause a wide range of impairments, including cognitive, sensory or motor impairments. Some studies have considered the use of tDCS for non-motor impairment [222, 223], but evidence of tDCS for motor neurorehabilitation after TBI is currently lacking [224–226]. We found one tDCS-study that included trauma-injured conditions (TBI and stroke) focusing on motor recovery. Motor improvement was reported after 24 sessions of bihemispheric tDCS over motor primary cortex (electrode size: 2 × 2 cm; 1.5 mA; 15 min; 24 sessions (3 days/week); the anodal electrode placed over the ipsilesional M1 and the cathodal electrode over the contralesional M1; Fig. 4b) associated to physical therapy [143]. This preliminary human result and some from animal studies [119, 227] have supported the potential benefit and safety of DCS after TBI. However, the diffuse damage associated with TBI, making it difficult to determine the stimulation target, could limit the use of tDCS as a therapeutic modality to improve motor outcomes after TBI.

## tDCS as a motor neurorehabilitation tool in musculoskeletal disorders

Musculoskeletal disorders involve a set of diseases coming from skeletal, articular and muscular systems, and associated with dysfunction from the cellular to biomechanical levels. Plasticity in the brain, however, has been often neglected in people with musculoskeletal disorders, and may be a factor influencing disease initiation and maintenance. Reorganization of the motor cortex has been described in many musculoskeletal conditions (see below). Unfortunately, current evidence involves the combination of musculoskeletal disease and pain, generally chronic pain (CP), making it difficult to disentangle those conditions to understand whether reorganization is related to the musculoskeletal disorder per se, or to CP.

Neurophysiological changes associated with musculoskeletal dysfunction associated to CP have been studied with TMS. A review of studies on migraine, musculoskeletal and neuropathic pain has reported no difference in resting motor threshold and motor evoked potential (MEP) between people with or without CP [228]. When only TMS studies on musculoskeletal pain are individually analyzed, MEP is reported to be higher in low-back and patellofemoral pain, but not neck pain, fibromyalgia, arthritis or myofascial pain [228]. TMS motor maps, however, are consistently rearranged. Previous studies have demonstrated changes in the primary motor muscle representation in different conditions. Low back pain, for example, is related to a decrease in the multifidus cortical map size which also has its center of activity (Center of Gravity) superposed with the superficial erector muscles [229, 230]. This abnormal representation may be associated with muscle activation dysfunction, altered activation/coordination of tonic/phasic muscles, and impaired biomechanical patterns of movement. The same has been shown for the quadriceps muscle in people with knee pain [231] and the extensor digiti muscles of people with tennis elbow [232]. Those changes in the motor maps might constitute key factors in sustained muscle pain [233] and have been associated with disease severity [229].

Therefore, TMS findings from musculoskeletal dysfunction indistinguishable from the presence of CP include a variable increase of MEP and motor maps rearrangements in the M1. These findings characterize a state of maladaptive plasticity, where changes in the central nervous system organization and functioning lead to decreased function through abnormal sensorimotor activity and pain.

### tDCS as a tool to counteract maladaptive plasticity in chronic musculoskeletal pain

Electrical currents can be used to modulate pain in various manners. One of its uses is by applying them directly on the scalp of cortical brain structures to modulate neural networks, inhibiting or stimulating endogenous brain activities (in sessions of 20 to 30 min and for at least five to ten consecutive days). They can also be applied to peripheral (extra cephalic) anatomic structures with the aid of surface or needle electrodes applied to the skin.

Though Peripheral Electrical Stimulation (PES) is not the topic of the present review, the combination of tDCS with PES has gained prominence as a method to potentiate the effects of tDCS. The application of monophasic or biphasic currents in musculoskeletal regions and/or over nerve trunks of peripheral nerves within less than 30 min at the sensory threshold and with frequencies ≧10 Hz produces an inhibition of intracortical synapses [234]. On the other hand, stimulation performed at the sensitive threshold or low level of motor activation that promote weak contractions without fatigue can increase cortical excitability [234, 235]. In this last case, electrical currents should be applied for 60 to 120 min (usually 90 min) with frequencies < 10 Hz. As such, depending on the duration, current intensity and frequency, PES can also modulate the M1, producing analgesia. The combination of excitatory a-tDCS with inhibitory sensory PES has been shown to potentiate the effects of the first, although the exact mechanisms associated with this combination are unknown [236, 237]. Hence, tDCS associated with PES may be used effectively in the control of CP associated with musculoskeletal and neuropathic conditions.

The most commonly used targets for neuromodulation of musculoskeletal dysfunction and CP are the M1 (electrode size: 5 × 7 cm; 2 mA; 20 min; anodal C3/cathodal Fp2; 5 sessions; Fig. 4a) and left DLPFC [18, 138]. Previous work with TMS has demonstrated that stimulation of these regions modulates pain-related areas via the activation of dopaminergic, glutamatergic, adrenergic, and cholinergic pathways [238]. In the 90’s, a group of Japanese neurosurgeons were seeking targets for implants in the cortex and recording thalamic activity while doing cortical stimulation to look for cortical areas related to pain in cats [239]. At first, they expected the S1 to be the best target. However, since they were next to the M1, they tested it by chance. They observed that there was decreased thalamic spike activity only when they stimulated the M1 and there was fairly no response to the stimulus of the S1. Later on, this cortico-thalamic pathway has been shown to be involved in the modulation of the grey periaqueductal substance, the cingulate cortex, and indirectly the amygdala, primary and secondary S1, spinal cord, and trigeminal ganglion [240]. Thus, the M1 is a good target for neuromodulation because it reaches a wide network related to pain control. a-tDCS applied to the M1 can probably control pain through the restoration of cortical modulation of the pain network [241]. It also seems that the stimulation has a somatotopic effect, that is, the closer to the cortical painful representation, the better the result [242]. However, the M1 stimulation also has a diffuse analgesic effect, and diffuse pain syndromes may be treated by this technique [243, 244].

The prefrontal cortex, another target for modulation of pain, is an executive area and directly influences the M1. This area is dysfunctional in CP [245], suggesting that its modulation would be a relevant goal. DLPFC stimulation has the potential to promote pain control, as it modulates the M1 and is dysfunctional in CP patients. However, its effectiveness in promoting analgesia has been refuted in many studies [92, 246, 247], although it would be interesting in the control of relevant aspects associated with CP such as decreased cognitive performance and depression.

a-tDCS of the M1 to treat CP patients has been validated in different pain syndromes such as fibromyalgia, neuropathic pain, and musculoskeletal pain, among others [18, 138]. The European Federation of Clinical Neurophysiology has attributed a level C of recommendation in the treatment of lower limbs pain associated with spinal cord injury and a level B in the treatment of fibromyalgia [92]. However, a comprehensive meta-analysis has shown that a-tDCS over the M1 has only a minimal clinical effect in the control of pain, but a consistent impact on increasing quality of life in chronic pain patients [247]. A recent consensus recommended as a level A for a low (from 20 to 30%) to moderate (from 30 to 50%) benefit in the control of pain associated to fibromyalgia; a level B recommendation for neuropathic pain, abdominal pain, musculoskeletal pain, and migraine and a level A of recommendation against the use of a-tDCS in the M1 alone in low treatment of low back pain [138]. The combination of a-tDCS in the M1 with sensory PES at the painful area has been shown to be a way of potentiating the effects of tDCS. Schabrun et al. [237] showed that this combination was the most advantageous in decreasing pain in increasing multifidus M1 map volume (a measure of the total excitability of the cortical representation) in patients with low back pain. Hazime et al. [248] found similar results, showing that a-tDCS over M1 associated with 100 Hz sensory PES lead to a greater clinical effect than tDCS and PES alone or sham stimulation.

## Facilitation of motor learning and consolidation by tDCS in patients and athletes

Motor control refers to the process of achieving a desired coordinated movement by the nervous system structures. Motor cortex projections to motor circuits within the spinal cord are closely linked to muscle control [249]. Motor learning depends on the motor cortex to learn new movements, anticipate or adjust the desired action [250]. Motor cortex learning-related plasticity involves synaptic strength [251] and dendritic spine growth [252]. Stabilization of these modifications involves intracellular signal transduction cascades, neuronal protein synthesis, and neural networks [253]. A fundamental question arises as to how tDCS modulates neuronal polarization. The low current up to 2 mA through non-invasive electrodes on the scalp [35] modulates the neuronal excitability accordingly with electrode polarity. In general, it is assumed that anodal stimulation current enters the tissue inducing excitatory effects, and cathodal stimulation current exits the tissue inducing inhibitory effects. Anodal stimulation increases neuronal firing rates and intracellular Ca^+ 2^ concentration [60] which is possibly related to long-term potentiation mechanisms [254]. Whereas tDCS biophysics effects modulate neuronal membrane polarization [255], a second question then arises whether tDCS benefits motor learning on rehabilitation and sports performance. Nitsche and Paulus [43] demonstrated a polarity-dependent modulation of motor cortex excitability with tDCS (up to 1 mA, anode motor cortex, cathode forehead above the contralateral orbitofrontal). MEP amplitude of right abductor digiti minimi muscle was higher after anodal stimulation. As the MEP amplitude is related to the corticospinal excitability [256], tDCS appears to be effective to modulate motor learning in both, health and disease conditions. Several studies have reported anodal M1 stimulation related to behavior improvement, such as executive function and rowing performance [257], self-perception, but not swimming performance [258], learning novel skill [33, 139, 259], isometric contraction [260], countermovement jump performance test [261], motor imagery and finger tapping reaction time (RT) in elderly [262], and cognitive and visual attention performance [263]. Regarding motor learning (electrode size: 5 × 5 cm; 1 mA; 20 min; 5 sessions; Fig. 4a), the anodal electrode has been placed over a presumed “target” (eg.: left M1 to target right upper limb, C3) with the cathodal electrode located over the contralateral supraorbital region (eg.: right supraorbital area, Fp2) (Reis et al. 2009). However, the tDCS biophysics effects on the nervous system is beyond the M1. Shimizu et al. (2017) used anodal cerebellar tDCS, and showed enhanced transfer performance on fine motor sequence learning and generalization. On the other hand, Foerster et al. (2017) showed that cathodal cerebellar tDCS impaired static balance [264]. As these behavior changes depend on the tDCS biophysics (polarity, current, time) and neurophysiology (brain target, function, connection), such neuromodulation method is challenging human limits. In spite of improving learning and motor performance, tDCS also has a boost effect when it reduces fatigue perception [21, 265–267].

Therefore, the tDCS effects shown in this growing range of protocols exploring intensity, dosage and electrode assembly [33, 259, 268–271] are supporting new approaches, not only to sports [259], but to promote physical and cognitive rehabilitation in several pathological conditions [32, 33]. The rationale, for example, is to use anodal tDCS to stimulate the lesioned motor cortex or cathodal tDCS to inhibit the contralateral motor cortex, and improve motor learning and motor skills [272].

Another aspect of motor learning which athletes excel on is timing. Learning when to perform an action (and when to withhold responding), and doing so with precision (i.e., with as little variability as possible) is important in virtually any sport modality. Although the number of studies focusing on temporal aspects of motor learning is still small, there is some evidence for the beneficial effects of tDCS on motor timing. For example, Arias et al. [273] tested whether stimulation of M1 improved performance in a fast arm reaching task. In this procedure, healthy participants had to reach for an object as fast as possible after a signal (auditory cue) was presented. The results showed a premotor reduction time after either anodic or cathodic stimulation of M1. That is, the time between signal and movement-related EMG onset decreased compared to sham stimulation. Moreover, the authors showed that fatigability (i.e., increased reaching times when the trial was repeated) was also avoided by real stimulation.

Very few studies have also shown that non-invasive brain stimulation (NIBS) may improve temporal processing in larger temporal scales, in the range of seconds to minutes (referred to as *interval timing* by the timing community). Mainly, these studies have suggested that tDCS over the posterior parietal cortex (PPC) enhances temporal discrimination [274–276]. Moreover, when this area is disturbed by transcranial random noise stimulation (tRNS), temporal performance is disrupted [277]. Finally, stimulation of DLPFC [148] and primary auditory (A1) and visual cortices (V1) [149] also seems to affect temporal performance. Despite these promising results, the effects of tDCS on temporal performance are still largely unknown, but they may contribute to the understanding of the neural basis of timing.

## Other effects of motor areas modulation

### Underappreciated motor cortex stimulation for psychiatric disorders

The motor cortex is usually not tDCS as the first target for most psychiatric disorders. In fact, the prefrontal cortex is usually stimulated for depression and schizophrenia [278–280]. Notwithstanding, the motor cortex role in psychiatric disorders might be underappreciated according to evidence from motor cortical excitability studies in these disorders. For instance, in a study involving 60 patients with major depressive disorder and 21 controls, patients presented decreased cortical silent period values as a measure of cortical inhibition compared to controls. In addition, atypical depression presented a distinct cortical excitability pattern characterized by decreased cortical inhibition and increased cortical facilitation compared to other depression subtypes [281]. In fact, a meta-analysis investigating motor cortical excitability in psychiatric disorders showed that inhibitory deficits are a ubiquitous finding across major psychiatric disorders and enhancement of intracortical facilitation is specific to obsessive-compulsive disorder [282].

There is also evidence that stimulation of non-motor areas affects motor cortical excitability in psychiatric disorders. In schizophrenia, a recent study performing non-motor, sham-controlled, double-blinded tDCS (anode and cathode positioned over the left prefrontal and temporoparietal junction, respectively) found changes in cortical inhibition after active but not sham tDCS [283]. This is not surprising as electric current simulation models show that under this tDCS montage motor cortical areas are also activated [184]. Particularly, tDCS might be an interesting option in patients with schizophrenia and prominent motor symptoms such as catatonia [285, 286].

Moreover, motor cortical excitability assessments at baseline might be useful to predict antidepressant response of tDCS, as, in a large clinical trial in depression, it was found that lower intracortical inhibition values (increased GABAA-mediated inhibition) at baseline were associated with lower depression improvement for anodal - left / cathodal - right dorsolateral prefrontal cortex stimulation [12]. This is interesting as it suggests that motor cortical excitability is a biomarker for antidepressant response, further unveiling the role of motor cortex in depression and antidepressant response.

Finally, tDCS treatment for obsessive-compulsive disorder directly targets the SMA, as this brain area is involved in dysfunctional thalamic-cortical circuits related to obsessive-compulsive disorder pathophysiology. Promising results were observed in a pilot study investigating the efficacy of cathodal vs. anodal stimulation of SMA in 12 patients with obsessive-compulsive disorder [147]. The results have shown that cathodal stimulation of SMA (electrode size: 5 × 5 cm; 2 mA; 20 min; 10 sessions; Fig. 4e) for treatment-resistant obsessive-compulsive disorder [147]. In fact, a larger, randomized, sham-controlled trial investigating the efficacy of cathodal tDCS over the SMA (electrode size: 5 × 5 cm; 2 mA; 30 min; 20 sessions; Fig. 4e) in 44 patients with obsessive-compulsive disorder will help further clarifying the involvement of motor cortex in obsessive-compulsive disorder pathophysiology and clinical response [146].

### Language and embodied cognition

Theoretical advances in cognitive neuroscience, particularly regarding the neural instantiation of language, emphasize the embodied nature of human cognitive functions. In this regard, the effects of modulating motor networks activity (e.g., using tDCS) on language provide an important framework for testing embodied theoretical cognition models. The M1, for instance, is arguably enrolled in functions extending far beyond the mechanical implementation of motor programs, which includes high order functions such as memory [287] and the processing of action-related abstract concepts [288]. Both passive listening and categorization of verbs referring to upper or lower reliably reduce corticospinal excitability in a somatotopic fashion, according to limb recruited by the verb [289]. In a single pulse TMS study, the amplitude of MEP in the leg and arm muscles were shown to be selectively modulated in a categorization task of learned names of soccer or tennis players [290]. Crucially, words arbitrarily associated with tennis categories seemed to be sufficient to modulate corticospinal representation of leg muscles, reinforcing that M1 is involved in processing abstract action-related concepts. In a related study on the role of the M1 in speech perception, a-tDCS, c-tDCS or sham tDCS was applied to the left M1 during a task of picture recognition simultaneously presented with a sentence, both with or without motor content [145]. c-tDCS (electrode size: 5 × 7 cm; 2 mA; tDCS started 4 min before the beginning of the task and was delivered for the whole course of the task execution, about 2 min; the cathodal electrode positioned over the left M1 and the anodal electrode placed on the skin overlying the left shoulder region; Fig. 4d) has shown to improve the detection of mismatches between a motor and non-motor sentence/picture associations. This result provided further evidence for the role of motor areas in semantic processing of action verbs. The processing of the meaning of action verbs also seems to be correlated with PMC activity. Differential excitation and inhibition of these areas using a-tDCS and c-tDCS over bilateral PMC in the two possible montages before a lexical decision task showed complementary effects: a-tDCS over the left PMC impaired performance in judging unimanual actions while c-tDCS improved performance (electrode size: 5 × 7 cm; 2 mA; 20 min; the cathodal placed at FC3 and the anodal at FC4; Fig. 4c) [144]. On the other hand, the motor learning of speech production has also been shown to be facilitated by tDCS modulation of motor areas [291]. Overall, these studies and results exemplify how tDCS has been used to test the degree of superposition between language and motor networks, contributing to other lines of evidence for the embodied cognition accounts of both language comprehension and production.

Although most principled studies applying embodied cognition and tDCS to date have focused on language, these theoretical premises imply that other cognitive and emotional domains might be influenced by motor networks modulation. tDCS applied over the right PMC have been used to test the neural basis of a body ownership illusion, namely the rubber hand in which a fake hand is perceived as part of the body [292]. a-tDCS increased the intensity of the illusion, with a greater misperception of the position of the real hand to the fake one. As pointed out by the authors, the possibility of understanding and influencing body ownership experiences using tDCS might improve treatment and rehabilitation in different neurological conditions. Particularly, the improved neural representation of prostheses should improve patient’s functionality.

### Functionality and social aspects

The main objective in neurorehabilitation is on the rapid establishment of independence in activities of daily living (ADLs) through compensatory strategies [293]. Functional impairment after injury could result in poor performance in ADLs and social impairment [130]. A proper approach in the neurorehabilitation practice encourages the use of the WHO International Classification of Functioning, Disability, and Health (ICF) [294, 295]. ICF is a universal framework and an international instrument for describing all aspects of disability [295]. According to this model, human (and individual) experience of functioning is not considered as the consequence of a disease, but the result of the interaction between a health condition and both personal attributes and environmental influences (social and contextual factors) [296]. Therefore, the ICF is a biopsychosocial approach that incorporates health components at the physical and social levels [296].

The challenge of neuromodulation is how to apply the ICF for rehabilitation management in clinical practice. tDCS could be part of a rehabilitation plan that comprises four steps: assessment, goal setting, interventions and outcome measurement [295]. ICF can be used as a reference instrument and framework to define interventions to promote motor rehabilitation and motor learning. Studies with tDCS demonstrate an improvement in motor performance and motor learning in general practice for healthy volunteers and patients suffering from neurological disorders [259, 268]. Almost in their entirety studies with healthy subjects or patients, the effects of tDCS have been reported for motor tasks such as serial RT tasks, adaptation tasks, or visuomotor tracking [32, 259]. However, for the new model of rehabilitation and inclusion, it is necessary to study functional measures of ADLs and the social aspects that tDCS can provide.

Disability often leads to reduced social participation, regardless of physical or cognitive limitations [297]. Social aspects including participation restrictions were evaluated using questionnaires and scales about the success and difficulties to do exercise, go to church or visit a friend [297]. The functional mobility concept considers how an individual moves daily through the environment to achieve successful interactions with family and society [298]. Studies with tDCS and motor rehabilitation presented interesting neurophysiologic data and patient symptoms but few studies investigated the relationship about motor improvement and daily living or social aspects. Floel [16] shown a summary statement on the present use of tDCS in the treatment of neurological disorders. None of the forty-six studies listed showed the effects of tDCS on motor and cognitive function associated with social aspects [16].

Elsner et al. [299] described in a systematic review about Parkinson that tDCS may improve impairment regarding motor symptoms and ADLs. After investigating six trials with a total of 137 participants, none of these studies describe the effects of tDCS on improving social aspects. Improvement in ADLs in people after stroke treated with tDCS was found in nine studies with 396 participants [130]. The authors found very low to moderate quality evidence of effect regarding ADLs performance at the end of the intervention period. Besides, no information about social aspects was found in this study [130]. For the ICF model, it is important a comprehensive overview of the patient functioning by presenting the assessment results in all components of human functioning [296].

To the best of our knowledge, no studies were found in motor rehabilitation with tDCS and ICF. There are several advantages pointed with the ICF model. We can highlight the possibility of standardization of concepts and, therefore, the use of a standard language that allows communication between researchers, managers, health professionals, civil society organizations and users in general [294, 296]. Besides, the ICF can be alternatively used to many sectors that include health, education, social security, labor medicine, statistics and public policies [294–296]. Studies about tDCS and motor rehabilitation could evaluate not only body functions but an integrative model of functioning, disability, and health that involve tasks of involvement in a life situation, environmental factors with social and attitudinal situations.

The use of ICF in neuromodulation practices comprise the incorporation of new technology, already adopted by several sectors and multidisciplinary teams. ICF should be widely explored in relation to its acceptability and validity including the impact on health care, the potential in measuring the functional status of patients and their use by information systems for the elaboration of health statistics [295]. ICF could be used to improve the legislation and the implementation of public policies in neuromodulation for people with disabilities.

Although ICF has become a universal standard in the neurorehabilitation process, there is still no integration of this process into clinical routine and scientific research involving tDCS. In general, however, it is clear that ICF has many advantages in the process of rehabilitation, allowing the elaboration of rigorous research projects and the achievement of results that demonstrate its value and potential.

## Future perspectives

### HD-tDCS on neurological disease, pain relief, and motor learning/rehabilitation

The so-called “conventional” tDCS, which uses large electrode pads involved in sponges embedded with saline solution, apply a diffuse electrical current to the brain which stimulates not only the target area but also unwanted regions in a non-predictable fashion. This presents a significant limitation given the low precision of stimulation (focality), which makes it difficult to discern which area contributed to the outcomes. In this regard, HD-tDCS uses a series of small electrodes over the target which circumscribes the stimulation to the diameter of the electrodes and presents improved focality as compared to the conventional tDCS (for a specific view of the HD-tDCS technique see [300]. Datta et al. [37] showed that a ring electrodes HD-tDCS montage (4 × 1) provided gyri precise stimulation while tDCS using electrodes pads (7 × 5 cm) resulted in a diffuse electric field (Fig. 2). Interestingly, the peak electric field was found to be not underneath the active electrode in the conventional tDCS, as it is usually presumed, while the HD-tDCS resulted in peak electric field at the sulci and gyri underneath the active electrode [37]. Experimental evidence has suggested that HD-tDCS may induce superior results compared to conventional tDCS [34]. For instance, Kuo et al. [34] compared the effects of conventional tDCS (electrode area 35 cm^2^) to HD-tDCS (4 × 1 ring configuration) using 2 mA for 10 min on corticospinal excitability, using MEP, in healthy participants. They showed that HD-tDCS induced greater modulation in MEP and this effect lasted longer than conventional tDCS (i.e., more than two and less than 6 hours) [34]. Interestingly, HD-tDCS presented a delayed peak effect magnitude, which occurred 30 min after tDCS [34]. These present important implications for the use of tDCS during training/therapy, given that the effect of conventional tDCS seems to decrease linearly over time. Thus, HD-tDCS represents a recent advance in NIBS considering that it overcomes the limitation of conventional tDCS. So far, however, few studies have compared whether this increased focality promoted by HD-tDCS could result in more significant improvements in the outcomes. This could be mainly because this technology is relatively new. tDCS has shown promising results for various neurological diseases [301–303]. For instance, Aleman et al. [302] conducted a meta-analysis of controlled trials and showed that NIBS of the frontal cortex improved negative symptoms of patients with schizophrenia, but the evidence for transcranial magnetic stimulation was stronger than for tDCS. Also, the existent literature supports the positive effects of a-tDCS on improving cognitive capacity in both healthy individuals and neuropsychiatric patients [301, 304]. Hogeveen et al. [305] compared the effect of HD-tDCS to three montages of conventional tDCS on response inhibition in healthy adults and found similar improvements for both forms of tDCS. On the other hand, Gozenman and Berryhill [306] showed that individuals with lower baseline working memory capacity benefited more from HD-tDCS than from conventional tDCS. In addition, an impressive result was presented by Trofimov et al. [307] who demonstrated that HD-tDCS (1 mA for 20 min) 21 days after a TBI reduced the number of areas with hypoperfusion and ischemia, increased cerebral blood flow, cerebral blood volume, and shortened mean transit time in 19 patients with TBI.

For some diseases/symptoms, however, there is still little evidence and the effectiveness of tDCS is uncertain. For instance, Elsner et al. [299] conducted a meta-analysis and concluded that there is insufficient evidence to determine the effect of tDCS on PD patients. A study by Dagan et al. [138] compared the effect of a single session of tDCS over M1 (single-target) and simultaneous stimulation of M1 and DLPFC (multi-target) using HD-tDCS on motor and cognitive function in PD patients. They found improvements in motor (i.e., reduced severity of freezing of gate, timed up and go performance, gait speed) and cognitive (i.e. Stroop interference test) performance only after multi-target stimulation [138]. This suggests that HD-tDCS targeting both motor and cognitive regions may be more effective than single M1 stimulation for PD. Studies using HD-tDCS for PD are scarce so that it remains relatively unexplored whether this technique could produce better results compared to conventional tDCS. Similar to PD, the extant literature does not support the efficacy of tDCS for treating auditory hallucinations, a common symptom of schizophrenia [308]. However, two recent studies used HD-tDCS for auditory hallucinations with promising results [309, 310]. Sreeraj et al. [309] applied HD-tDCS using the 4 × 1 ring montage with a cathode as the central electrode over CP5 (i.e. left temporoparietal junction) with 2 mA for 20 min, two sessions per day for five days on 19 schizophrenia patients and found a significant reduction in persisting auditory hallucinations. Similarly, a case series study in patients with dementia presenting severe auditory hallucinations suggested that HD-tDCS appears to be an effective treatment option [310].

Meta-analytical evidence has shown that a-tDCS over the S1 and M1 increase the sensory and pain threshold in healthy individuals [311]. Similarly, a-tDCS over M1 and DLPFC decreased pain levels in patients suffering from CP [247, 311, 312], which represent an improvement clinically significant, as well as in quality of life [247]. But only two studies with HD-tDCS were included [313]. Interestingly, it has also been shown in another meta-analysis that c-tDCS over S1 and M1 increased sensory and pain thresholds in healthy individuals and pain levels in patients [314]. Similar results were found by Villamar et al. [313] that showed both anodal and cathodal HD-tDCS reduced pain perception in patients with fibromyalgia. As previously presented, HD-tDCS presents improved focality in comparison to conventional tDCS. DaSilva et al. [315] tested a variety of tDCS montages targeting brain regions related to the pain processing used in studies involving migraine and pain control and compared conventional to HD-tDCS with high-resolution computational forward modeling. They showed that conventional tDCS montages presented large current flow and peaks of current flow often not at the target of stimulation, occurring in deeper brain regions, which in some cases were not even related to the outcome (e.g. visual cortex) [315]. On the other hand, HD-tDCS montages enhanced focality with peak current flow in subcortical areas at negligible levels [315]. Studies comparing conventional to HD-tDCS for pain have shown similar outcomes, however, for patients with tinnitus [316]. Remarkably, Castillo-Saavedra et al. [310] performed a phase II open-label trial aiming to define a treatment protocol for clinical treatment of pain in fibromyalgia using HD-tDCS. They found that both responders and non-responders similarly improved quality of life and decreased pain with a clinically significant pain reduction of 50% in half of the sample [317]. Finally, the authors estimated 15 sessions of HD-tDCS to reach clinically meaningful outcomes [317].

Regarding motor performance, a recent meta-analysis confirmed that a-tDCS increases corticospinal excitability of the M1 (i.e. MEP size), intracortical facilitation and decrease short-interval intracortical inhibition in healthy individuals [318, 319], which could implicate increased motor performance, but only one study using HD-tDCS was included [34]. Different studies have used tDCS for motor performance enhancement, with some showing positive results while others null results (see the meta-analysis by Machado et al. [320] for a detailed discussion on the effect of tDCS on exercise performance). Radel et al. [321] and Flood et al. [322] were the only two studies to test the effects of HD-tDCS (4X1 ring montage) on the time to task failure on a submaximal contraction of the elbow flexors and knee extensors, respectively, in healthy adults and showed no improvement. These results were confirmed in a recent meta-analysis that showed no significant improvement in isometric strength performance [323]. On the other hand, HD-tDCS (1 mA for 15 min) over bilateral M1 during motor training (3 days) improved unimanual and bimanual dexterity in healthy individuals, suggesting a positive effect on motor learning [324, 325]. However, these studies did not include groups receiving conventional tDCS to compare efficiency between both techniques. Similar results of motor learning were also shown with conventional tDCS (2 mA for 20 min) over the M1 applied during motor training (5 days) in healthy individuals [326]. In fact, meta-analytical evidence has confirmed that both single and multiple session of tDCS applied over the M1 improves motor learning in healthy individuals and post-stroke patients [133, 327]. So far, Cole et al. [328] performedthe only study comparing the effects of conventional and HD-tDCS (4X1) over the M1 on motor learning in a group of children. Participants underwent training over five consecutive days and were assessed at baseline, post-training and 6 weeks after training (i.e., retention). Both conventional and HD-tDCS similarly improved motor learning not only after training but also after 6 weeks as compared to the sham group [328].

In sum, HD-tDCS holds the promise to be more effective than conventional tDCS, though since it is a relatively new technique, there is a small number of studies using HD-tDCS, and especially, comparing both forms of stimulation. Soon, systematic reviews and meta-analytical studies may be able to compare outcomes between techniques to elucidate efficiency. So far, the results found for HD-tDCS are at least comparable to conventional tDCS.

## tsDCS on clinical applications

**Fig. 5:**
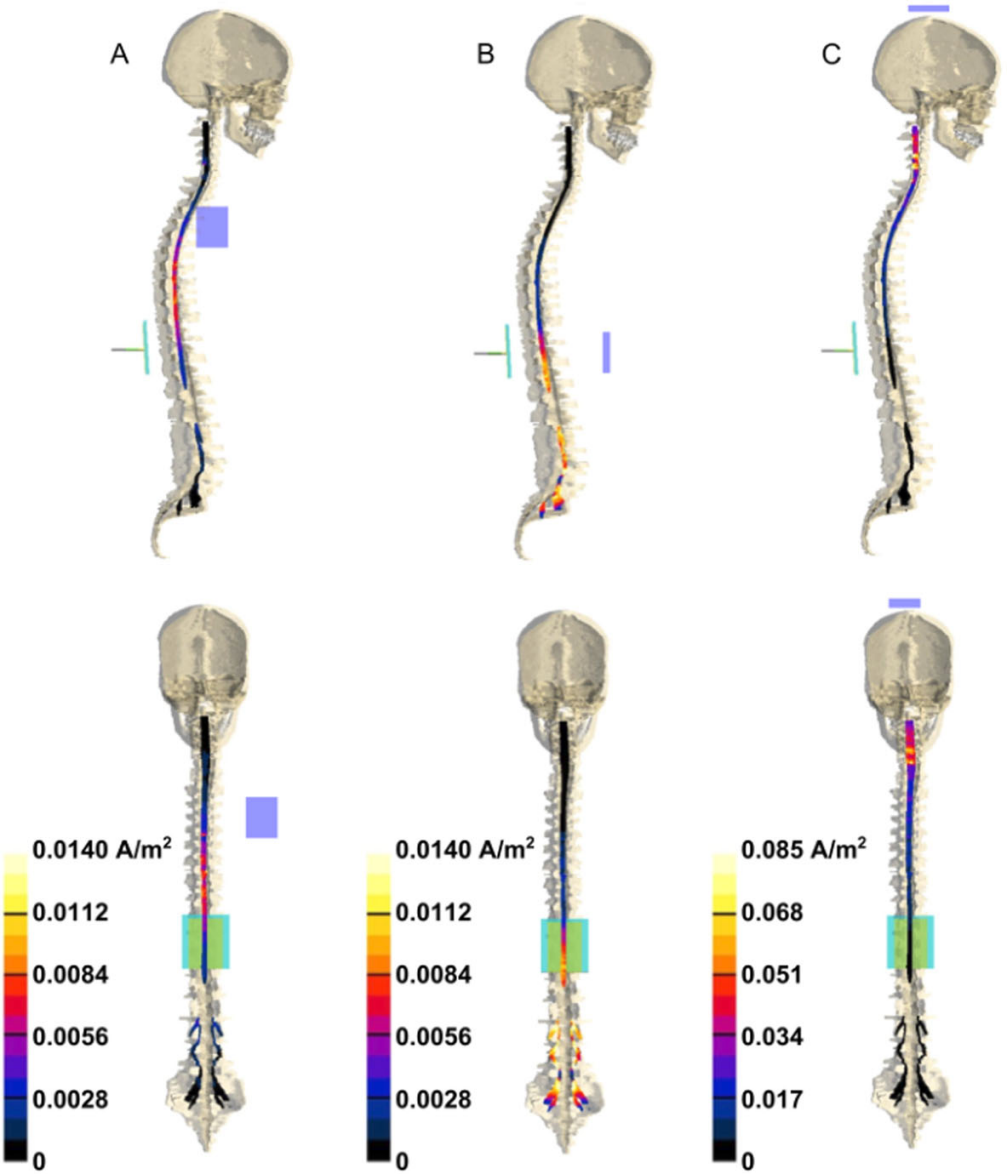
tsDCS electric field distribution in tissues. Lateral (1st row) and front (2nd row) view of the J amplitude distribution over spinal cord and nerves for three different montages: **a** (left column, return electrode placed over right shoulder); **b** (middle column, return electrode over abdomen); **c** (right column, return electrode at the vertex). Modified from Parazzini et al. [335], with permission

In recent years, current polarization of the spinal cord has emerged as a novel and promising method for modulating spinal and supra-spinal excitability. The so-called tsDCS has been assessed for the treatment of pain [329–331], spasticity [332], stroke [333, 334] and spinal cord lesions [207]. DCS intensity ranges from 1.5 to 3.0 mA, with effects lasting for minutes to hours [90]; the device is the same used for tDCS, although different authors have used electrodes of different sizes and with different montages (Fig. 5), thus critically influencing current density and distribution in biological tissues [335, 336].

A growing body of literature has shown that tsDCS combines spinal and supra-spinal mechanisms of action. The later prospect is particularly attractive; for instance, in spinal cord injury (SCI) and stroke, tsDCS may interfere with the maladaptive reorganization of cortical sensorimotor maps, improving motor output and possibly preventing central pain sensitization [334, 337, 338]. That implies that tsDCS could be useful also as an early rehabilitation strategy in patients with acute brain lesions, when other NIBS tools are commonly avoided due to safety concerns. Another advantage is that tsDCS shows both in-line and off-line effects, thus influencing task-dependent and task-independent neuronal plasticity [339–341].

tsDCS exerts polarity-specific effects opposite from those reported for tDCS: while anodal tsDCS has an overall inhibitory effect, cathodal polarization improves the conduction along the corticospinal tract, spinothalamic and lemniscal pathways [342–344]. At a spinal level, anodal stimulation acts directly on axons, without affecting postsynaptic motor neuronal excitability, whereas cathodal stimulation preferentially interferes with interneuronal networks [345–347]. Specifically, in agreement with its facilitatory action, cathodal tsDCS seems to improve motor unit recruitment in healthy individuals, likely through an inhibition of the Renshaw cells network [346]. Others have reported similar effects of anodal and cathodal tsDCS [348], probably due to the different protocols used or to the presence of genetic polymorphisms [349].

Studies have also shown supra-spinal mechanisms of action of tsDCS, both in animal [339] and human models [346]. In particular, studies have demonstrated tsDCS after-effects on intracortical GABA_(a)_ergic networks and interhemispheric processing of motor output and visual stimuli [350, 351]; accordingly, Schweizer and colleagues have recently shown that tsDCS modifies functional FC within the somatomotor system in a polarity-dependent manner [338]. These changes might be not only secondary to plastic alterations occurring at the level of stimulation, but also due to the direct modulation of ascending spinal pathways, especially to the noradrenergic locus coeruleus neurons which have widespread projections to the neocortical brain [352].

Finally, a novel and exciting mechanism of action has been recently proposed by Samaddar and co-workers [353]: they found that tsDCS also modulates the migration and proliferation of adult newly born spinal cells in mice, a cell population implicated in learning and memory; although the mechanisms are not fully understood, these findings suggest that tsDCS could be used, also in humans, as an early treatment to improve motor recovery in spinal cord lesions. In this connection, another study has confirmed that tsDCS increases locomotor skill acquisition and retention in healthy volunteers [354].

## ctDCS and influence on motor learning

The cerebellum drives motor learning phenomena and tDCS may offer an unique opportunity to study the involvement in these processes [187, 355–359]; in fact, despite interindividual differences, recent modeling studies have revealed that during ctDCS the current spread to other structures outside the cerebellum is negligible and unlikely to produce functional effects [360, 361].

**Fig. 6:**
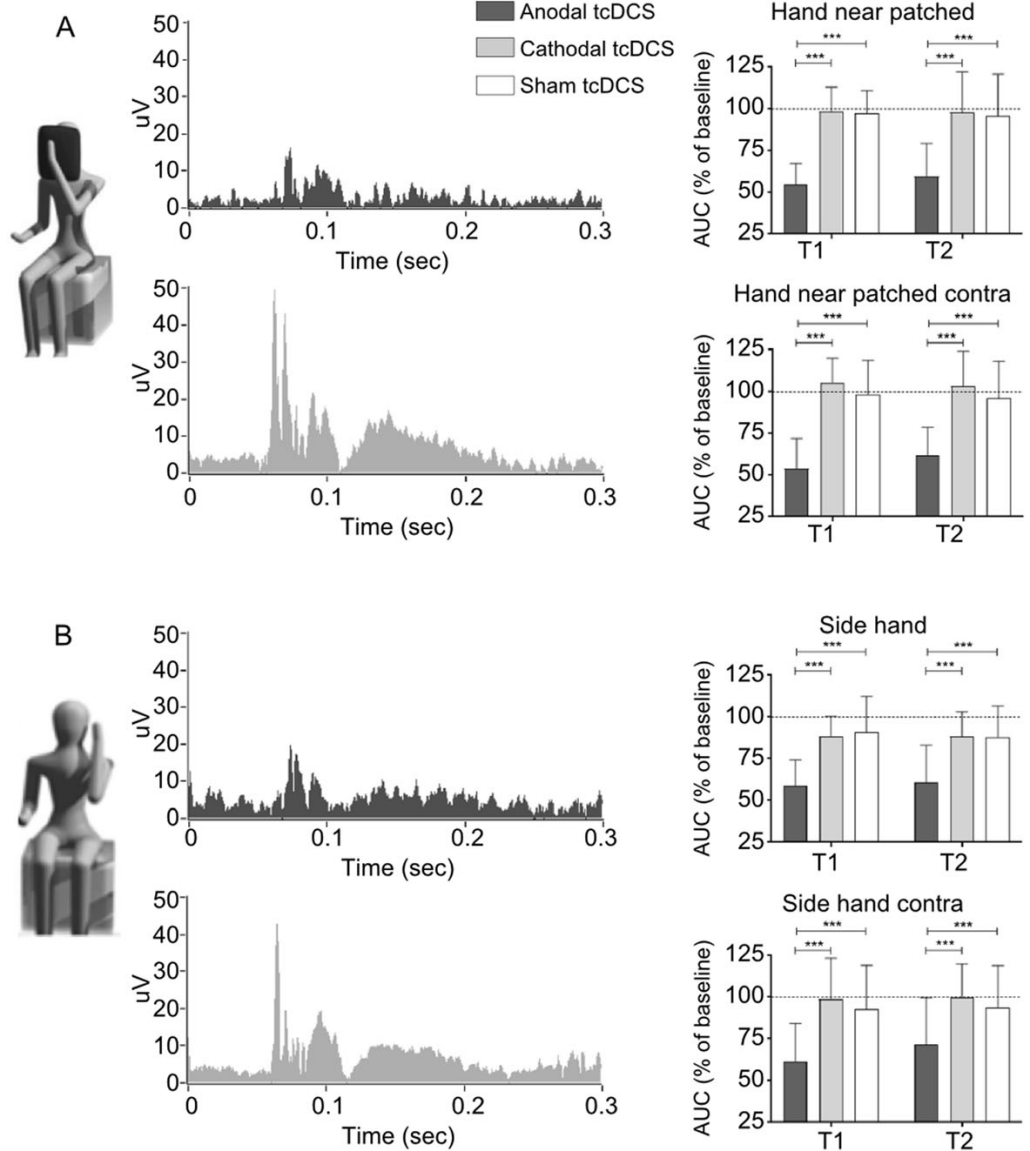
c-tDCS is able to modulate eyeblink conditioning, responsible for motor learning, as assesed by changes in Hand Blink Reflex (HBR) amplitude and area (experimental conditions: **a** patched hand; **b** hand side). Modified from Bocci et al. [369], with permission

From an historical perspective, the cerebellum and its related brainstem nuclei regulate the conditioned eyeblink response and contain long-term neuronal changes, which serves to encode this learned response [363–364]. The cerebellum is engaged in learning of unspecific aversive reactions and cerebellar dysfunction may lead to impaired short-term and long-term habituation of the startle response [365, 366], in agreement with the preeminent cerebellar role in encoding external negative stimuli [367, 368]. In a recent paper, Bocci and colleagues have shown that the cerebellum in also involved in motor learning finalized to defensive behavior within the peripersonal space [369] (Fig. 6).

Overall, by evaluating RT and error rate scores as clinical outcomes, several papers have recently demonstrated that excitatory anodal ctDCS enhances both on-line and off-line motor learning in healthy individuals [358, 370–372], probably by speeding up motor skill acquisition and accuracy (Cantarero [357] et al., 2015), particularly when combined with anodal tDCS over the primary motor cortex (M1; [351]. In this scenario, the cerebellum and motor cortex likely have distinct functional roles: whereas anodal ctDCS improves acquisition, as proved by a faster reduction of movement error, anodal M1 tDCS increased retention without affecting new motor skills acquisition [355]. Another study has shown that cerebellar stimulation does not affect the intermanual transfer of visuomotor learning, a key process in visuomotor adaptation and motor learning [373].

## TMS as a tool to evaluate tDCS effects on brain function

Proposed mechanisms for the therapeutic effects of tDCS include neurophysiological changes such as modified excitability, plasticity, neuronal oscillations, and connectivity between brain regions. TMS combined with EEG or Electromyography (EMG) is a powerful method that can be used to assess the integrity and modulation of such brain processes, and thereby evaluate the effects of a tDCS intervention [374, 375]. TMS excites the cortex non-invasively through a time-varying magnetic field induced by the application coil placed close to the surface of the scalp [376, 377]. Several TMS protocols have been designed using single and paired-pulse TMS applied to one or more brain regions (or peripherally) to trigger and evaluate the integrity of specific brain processes [374, 378].

**Fig. 7:**
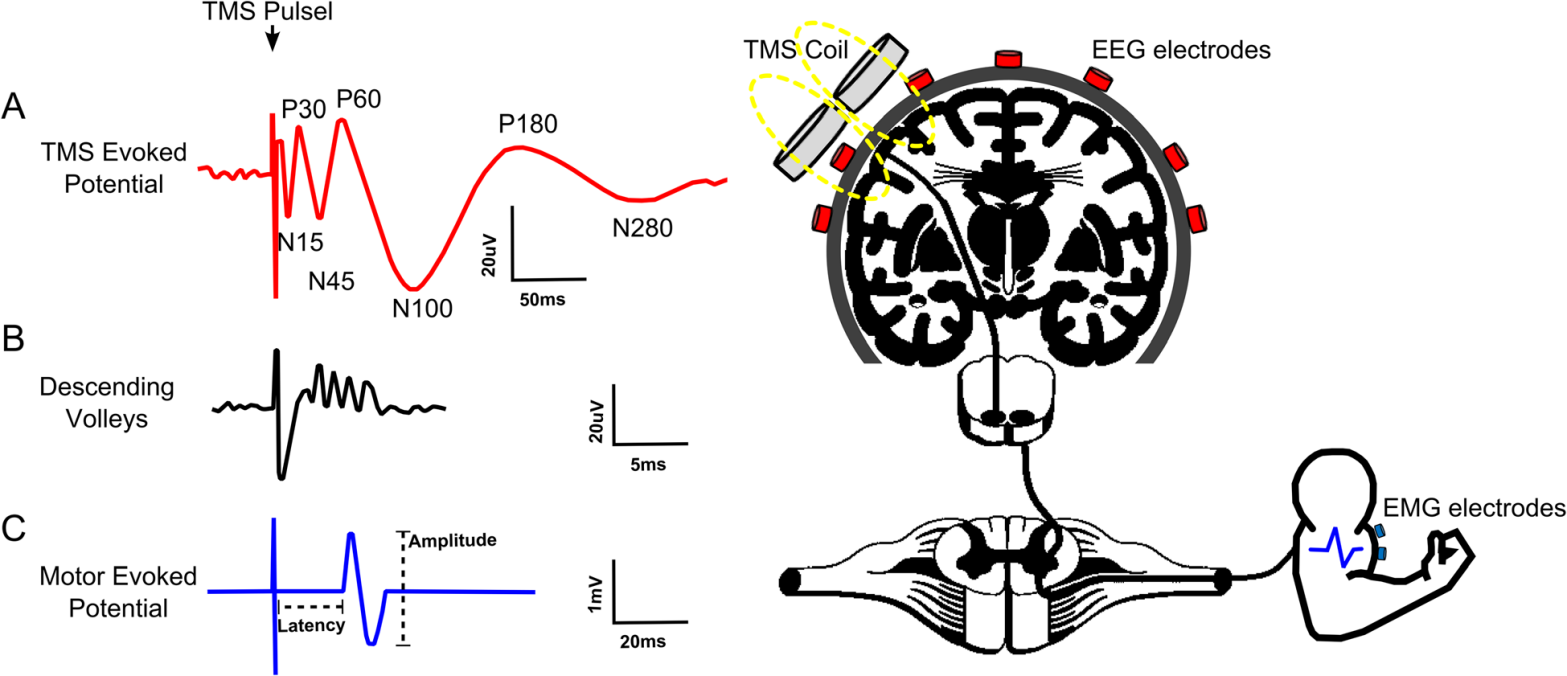
Illustration of TMS-induced evoked potentials throughout the nervous system, adapted from [374]. **a**) TMS pulse induces evoked potential detected by EEG recording. **b** TMS induced descending volleys in the corticospinal tract. **c** Motor evoked potential recorded by EMG

TMS-EEG can be employed before, after, and during an intervention to assess changes in brain circuitry and neurophysiology. Schematically shown in Fig. 7, TMS combined with concurrent EEG (TMS-EEG) can be used to measure local and global changes in brain reactivity and connectivity beyond the motor cortex. A TMS evoked potential (TEP) can be detected by EEG after a single pulse TMS. Different components of TEPs are linked to the activation of different brain processes. For example, earlier TEP components shown in Fig. 7a (e.g., positivity at 30 ms (P30)) are linked to excitatory mechanisms while later components (e.g., negativity at 100 ms (N100)) are linked to inhibitory processes [374]. In a few studies in patients with implanted electrodes, the impact of TMS on activation of corticospinal tract has been captured (Fig. 7b) and characterized as direct (D) and indirect (I) waves of descending volleys related to TMS induced activation of pyramidal and interneurons, respectively [379]. Finally, TMS applied to the motor cortex combined with peripheral EMG recording (TMS-EMG, shown in Fig. 7c) can characterize MEP or changes in EMG background activity. TMS-EMG can assess changes in corticospinal excitability through measures such as resting and active motor threshold, and cortical silent period (CSP), which are explained in detail elsewhere [378, 380].

Therefore, TMS offers a controlled input to the brain to study the integrity of various brain circuitry. TMS can be also targeted to a specific brain region or network using structural or functional neuronavigation with MRI, fMRI or EEG [381]. It has been shown that TMS evoked potentials from TMS-EEG are reproducible within individuals which suggests that the tool can be used in tDCS test-retest studies [382, 383]. In recent years, signal processing toolboxes including *TMSEEG* have been developed to standardize the process of TMS-EEG data cleaning and preprocessing, including removing TMS-induced artifacts [384], which assist in more widespread adaptation of this methodology.

TMS-EEG shows great promise in extracting markers of health in clinical populations [374, 385, 386], and in characterizing healthy and disease brain states [387]. In another general category of experiments, TMS can be used to interfere with neural processes, while EEG captures the effect of the intervention in comparison to a baseline state [374].

TMS-EEG has been used in the literature to assess the mechanism of action in tDCS interventions. For example, single pulse TMS-EEG has been used to investigate the effects of cortical excitability and connectivity by measuring changes in GMFAs and local TEPs following both anodal and cathodal tDCS [7, 388]. For tDCS applied beyond the motor cortex, it was found that anodal tDCS of the left DLPFC modulates cortical excitability in patients with disorders of consciousness [389]. In a study of tDCS for post-stroke aphasia rehabilitation, improvement in speech fluency was accompanied by modified TMS-EEG response in tDCS stimulated areas [390]. Using power spectra analysis from TMS-EEG data, it was shown that the beta and gamma band powers were modulated following HD-tDCS over the DLPFC [391].

TMS-EMG is a useful tool to study the effects of tDCS targeting the motor cortex. The crossed-facilitation (CF) effect refers to when MEPs in one relaxed arm are facilitated by contractions in the opposite arm. Using TMS-EMG to generate MEPs and CSPs, stimulation of the right primary motor cortex (M1) with HD-tDCS was shown to increase the effect of CF; possibly due to modulated interhemispheric connectivity [392]. Another study used E-field modeling with experimental TMS-EMG validation to find that only tDCS oriented orthogonal to M1 in the central sulcus can modulate TMS-induced MEPs [86]. Multimodal approaches combining transcranial electrical stimulation and TMS-EEG/EMG can lead a deeper understanding of the effects and neurological mechanisms of tDCS [375].

When using TMS-EEG in clinical populations and in tDCS studies, several factors should be carefully considered and controlled. These include morphometry (changes in evoked potentials with age), proper optimization of TMS parameters, and varied genetics of study participants leading to differences in neurological responses due to stimulation [378]. Reproducibility of TMS-EEG measures in clinical populations may be increased or decreased, possibly linked to disease-related changes in the brain structure and function, such as changes in neuroplastic mechanisms [393]. Furthermore, TMS produces a loud clicking noise upon application which results in non-transcranial auditory evoked potentials [394], and can cause peripheral somatosensory responses by stimulating extracranial tissue electrically. These additional pathways of TMS to generate TEPs highlight the need to control for the effects of multisensory stimulation [395, 396]. Guidelines and recommendations for how to control for these factors and how to run a TMS-EEG experiment can be found in details elsewhere [374, 387].

## Conclusion

There is increasing scientific evidence that tDCS modulates the brain to establish new patterns of activity and functional improvement in healthy and disabled individuals. As the mechanisms of action underlying tDCS neuromodulation are better understood and technologies become available, future research should focus on personalized tDCS protocols based on individual needs. In addition, the integration of NIBS with neuroimaging, particularly concurrent (online) integration, provides objective outcome measures and allows for the optimization of interventions. Therefore, additional clinical trials will help to elucidate the therapeutic role of tDCS on neurorehabilitation in clinical practice.

## Data Availability

Not applicable.

## Abbreviations

A1: Primary Auditory Cortex
ADLs: Activities of Daily Living
a-tDCS: Anodal Transcranial Direct Current Stimulation
CF: Crossed-Facilitation
CP: Chronic Pain
CSP: Cortical Silent Period
c-tDCS: Cathodal Transcranial Direct Current Stimulation
ctDCS: Cerebellar Transcranial Direct Current Stimulation
DCS: Direct Current Stimulation
DLPFC: Dorsolateral Prefrontal Cortex
EC: Effective connectivity
EEG: Electroencephalography
EMG: Electromyography
FC: Functional Connectivity
fMRI: Functional Magnetic Resonance Imaging
fNIRS: Functional Near-Infrared Spectroscopy
HD-tDCS: High-Definition Transcranial Direct Current Stimulation
ICF: International Classification of Functioning, Disability and Health
LTP: Long-Term Potentiation
M1: Primary Motor Cortex
MEP: Motor Evoked Potential
MS: Mutiple Sclerosis
NIBS: Non-Invasive Brain Stimulaton
PD: Parkinson Disease
PES: Peripheral Electrical Stimulation
PMC: Premotor Cortex
PPC: Posterior Parietal Cortex
rTMS: Repetitive Transcranial Direct Current Stimulation
S1: Primary Somatosensory Cortex
SCI: Spinal Cord Injury
SMA: Supplemantary Motor Area
TBI: Traumatic Brain Injury
tDCS: Transcranial Direct Current Stimulation
TEP: Transcranial Magnetic Stimulation Evoked Potential
TMS: Transcranial Magnetic Stimulation
tRNS: Transcranial Random Noise Stimulation
tsDCS: Transcutaneous Spinal Direct Current Stimulation
V1: Primary Visual Cortex
